# Supplementary material for: Diffusible fraction of niche BMP ligand safeguards stem-cell differentiation
Source: Nat Commun. 2024 Feb 7;15:1166. doi: 10.1038/s41467-024-45408-7 (PMC10850516; doi:10.1038/s41467-024-45408-7)
Supplement: Supplementary file 1 — Supplementary Information [file 41467_2024_45408_MOESM1_ESM.pdf]

Supplementary Materials for

Diffusible fraction of niche BMP ligand safeguards stem-cell differentiation

Sharif M. Ridwan[1#], Autumn Twillie[1#], Samaneh Poursaeid[1], Emma Kristine Beard[1], Muhammed Burak Bener[1] Matthew Antel[1], Ann E. Cowan[2, 3] Shinya Matsuda[4,*] and Mayu Inaba[1,*]

1. Department of Cell Biology, University of Connecticut Health Center, Farmington, Connecticut, United States of America
2. Richard D. Berlin Center for Cell Analysis and Modeling, University of Connecticut Health Center, Farmington, Connecticut, United States of America,
3. Department of Molecular Biology and Biophysics, University of Connecticut Health Center, Farmington, Connecticut, United States of America
4. Biozentrum, University of Basel, Basel, Switzerland

[*] Correspondence: inaba@uchc.edu, shinyamatsuda0423@gmail.com

[#] These authors contributed equally to this work.

The PDF file includes:

Table S1, Figures S1 to S6
Legends for Supplementary Movie S1, S2, Source Data, Supplemental Data1

Alleles	*mCherry-dpp**[1]	*GFP-dpp**[1]	*mGL-dpp**[1]	*mSC-dpp**[1]	*HA-dpp**[1]	*GFP-dpp*
Sources	CRISPR knock-in. Fereres et al., 2019	CRISPR knock-in. Gift from Thomas Kornberg	This study, Rasouliha et al., 2023	This study, Rasouliha et al., 2023	Matsuda et al., 2021	Matsuda et al, 2021
Phenotypes	Homozygous viable<br><br>Potential generation of non-tagged Dpp fraction*[2]	Homozygous viable<br><br>Potential generation of non-tagged Dpp fraction*[2]	Homozygous semi-lethal<br><br>Rescuable with *pPA dpp 8391/X*	Homozygous semi-lethal<br><br>Rescuable with *pPA dpp 8391/X*	None	Haploinsufficient<br><br>Partially rescuable with *pPA dpp 8391/X* (patterning defects)
Tag location	AA465	AA465	AA485	AA485	AA485	AA485
GSC phenotypes after expression of MT under hub driver (FasIIIGal4)	Not tested	Reduced pMad with mCD8-MT	Reduced pMad with mCD8-MT	Not tested	Reduced pMad with HAtrap	Not tested
De-differentiation phenotypes after expression of MT under hub driver (FasIIIGal4)	Not tested	Accelerated dedifferentiation with Nrv-MT trap	Accelerated dedifferentiation with Nrv-MT trap	Not tested	Not tested	Not tested

*[1] Alleles used in this study

*[2] Tags placed at AA465 may be cut out at the last furin processing site

Table S1. Comparison of fluorescent tagged *dpp* alleles.

Fluorescent tags are inserted after the indicated amino acid (AA) in *dpp* isoformE (NM_164488.2). Fereres et al., 2019 (54); Rasouliha et al., 2023 (57); Matsuda et al., 2021 (20).

mSC: mScarlet, mGL:mGreen Lantern.

Figure S1

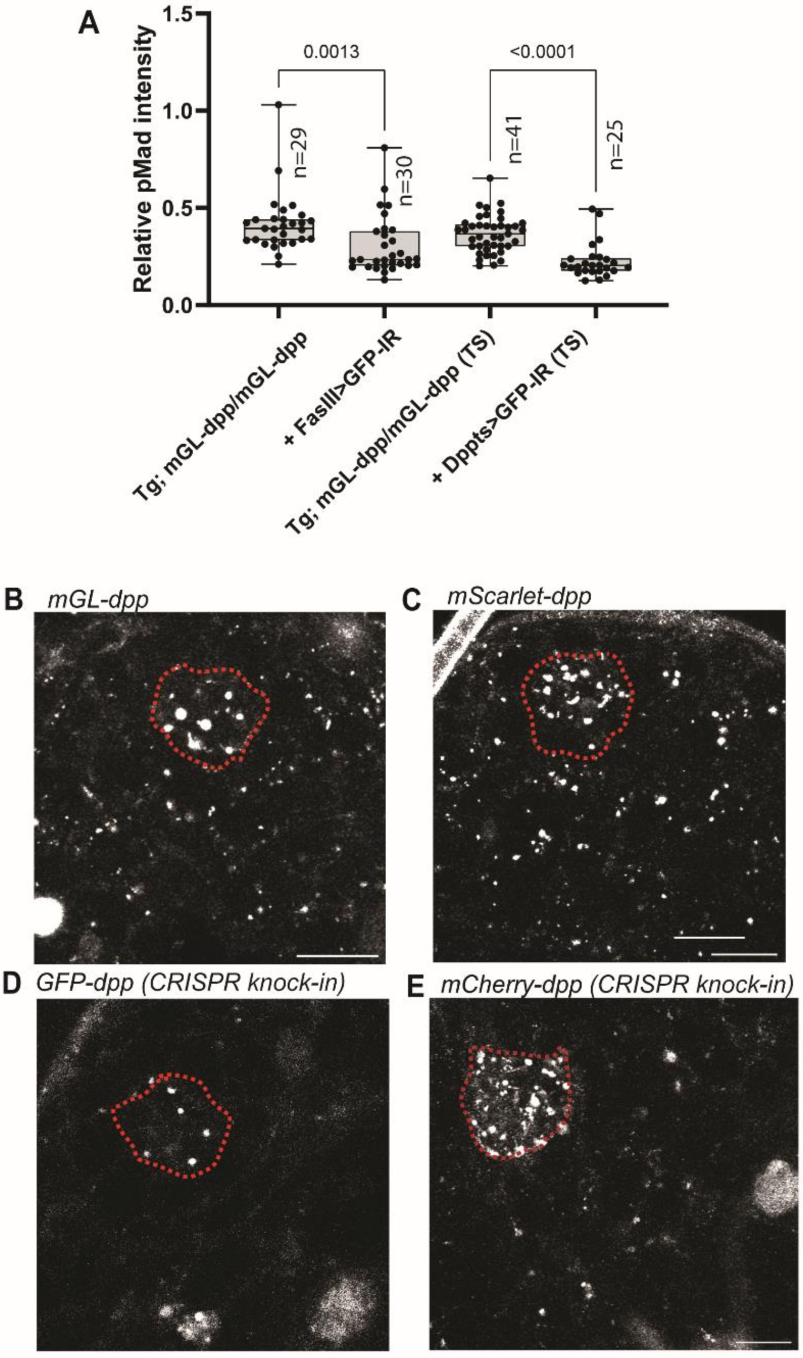
A
Relative pMad intensity
1.5
1.0
0.5
0.0
0.0013
<0.0001
n=29
n=30
n=41
n=25
Tg; mGL-dpp/mGL-dpp
+ FasIII>GFP-IR
Tg; mGL-dpp/mGL-dpp (TS)
+ Dppts>GFP-IR (TS)
B mGL-dpp
C mScarlet-dpp
D GFP-dpp (CRISPR knock-in)
E mCherry-dpp (CRISPR knock-in)

Figure S1. Comparison of fluorescent patterns of tagged *dpp* alleles.

A) Quantification of pMad intensity in GSCs (relative to somatic cyst cells or CCs) of indicated genotypes. fasIIIGal4 or dppGal4 (with tubGal80^{ts}) driven GFP (mGL) knock-down in homozygous *mGL-dpp* with *pPA dpp 8391/X*. Temperature shift (TS) was performed at 29 degrees for 2 days before dissection. p-values were calculated by Šídák's multiple comparisons tests and provided on the graph. Fixed samples were used. Numerical values are provided in Source Data file. “n” indicates the number of scored GSCs. Box plots show 25–75% (box), minimum to maximum (whiskers) with all data points. **B-E**) Representative images comparing testis tips isolated from homozygous *mGL-dpp* with *pPA dpp 8391/X* (**B**), homozygous *mScarlet(mSC)-dpp* with *pPA dpp 8391/X* (**C**), homozygous *GFP-dpp* CRISPR knock-in (**D**), and homozygous *mCherry-dpp* CRISPR knock-in (**E**). Scale bars represent 10 μm. The hub is encircled by red broken lines. Live tissues were used for imaging for **B-E**.

Figure S2

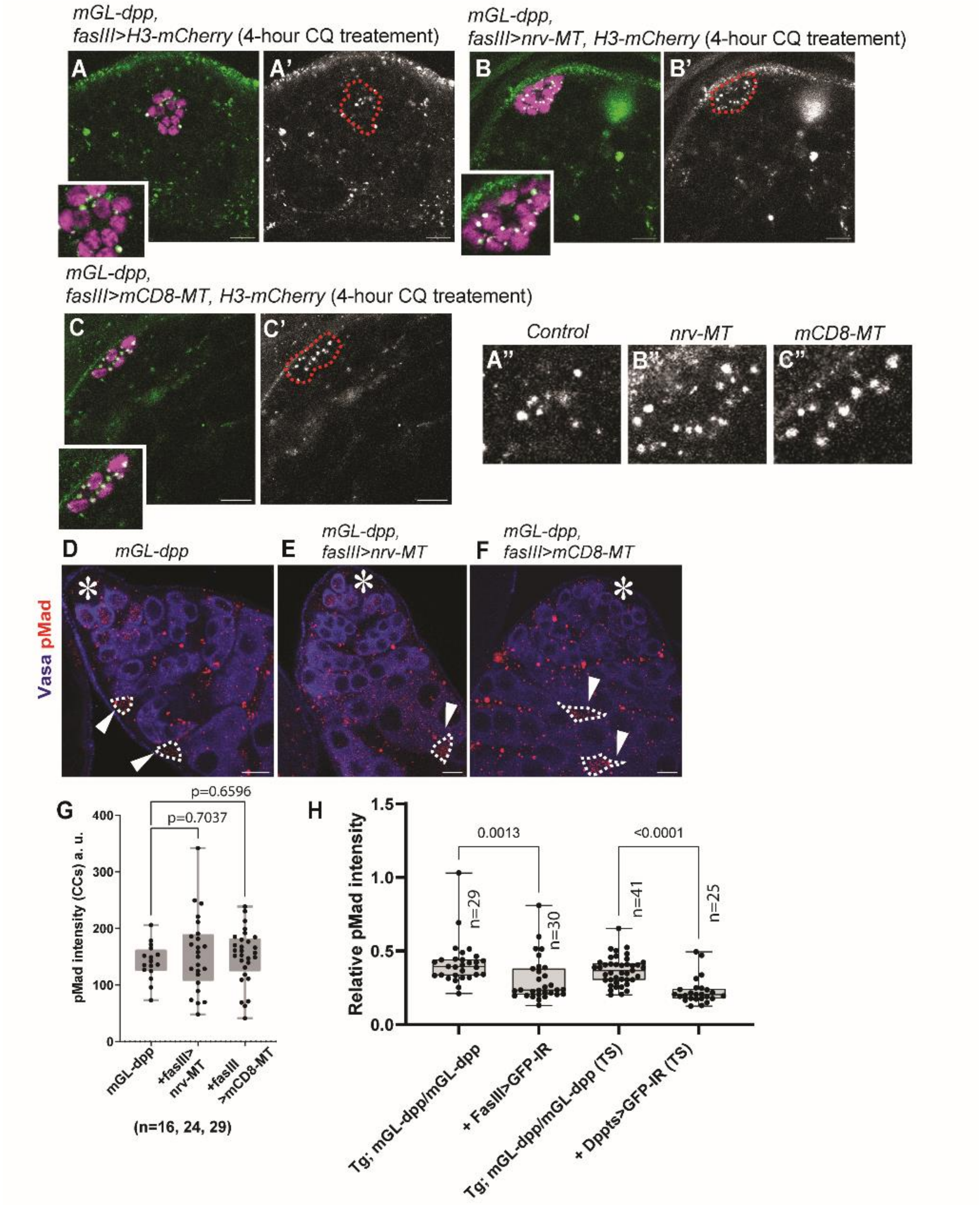
mGL-dpp,
fasIII>H3-mCherry (4-hour CQ treatement)
A
A'
mGL-dpp,
fasIII>nrv-MT, H3-mCherry (4-hour CQ treatement)
B
B'
mGL-dpp,
fasIII>mCD8-MT, H3-mCherry (4-hour CQ treatement)
C
C'
Control
nrv-MT
mCD8-MT
A''
B''
C''
D
mGL-dpp
E
mGL-dpp,
fasIII>nrv-MT
F
mGL-dpp,
fasIII>mCD8-MT
Vasa pMad
G
p=0.6596
p=0.7037
pMad intensity (CCs) a. u.
mGL-dpp
+fasIII> nrv-MT
+fasIII >mCD8-MT
(n=16, 24, 29)
H
Relative pMad intensity
0.0013
<0.0001
n=29
n=30
n=41
n=25
Tg; mGL-dpp/mGL-dpp
+ FasIII>GFP-IR
Tg; mGL-dpp/mGL-dpp (TS)
+ Dppts>GFP-IR (TS)

Figure S2. Characterization of morphotrap phenotypes.

A-C) Representative images of *mGL-dpp* signal after 4-hour CQ treatment in the testes of indicated genotypes. Magnified hub areas are shown in left corner of **A-C**. The hub is encircled by red broken lines in **A’-C’**. Magnified images of hub area are shown in **A”-C”**. Live samples were used. **D-F**) Representative images of pMad staining in somatic cyst cells (CCs) (marked by broken lines and arrowheads) of indicated genotypes. **G**) Quantification of pMad intensity in CCs of indicated genotypes. p-values were calculated by Šídák's multiple comparisons tests and provided on the graph. Fixed samples were used for **G**.

Asterisks indicate approximate location of the hub. All scale bars represent 10 μm. “n” indicates the number of scored GSCs. Box plots show 25–75% (box), minimum to maximum (whiskers) with all data points. Numerical values are provided in Source Data file.

Figure S3

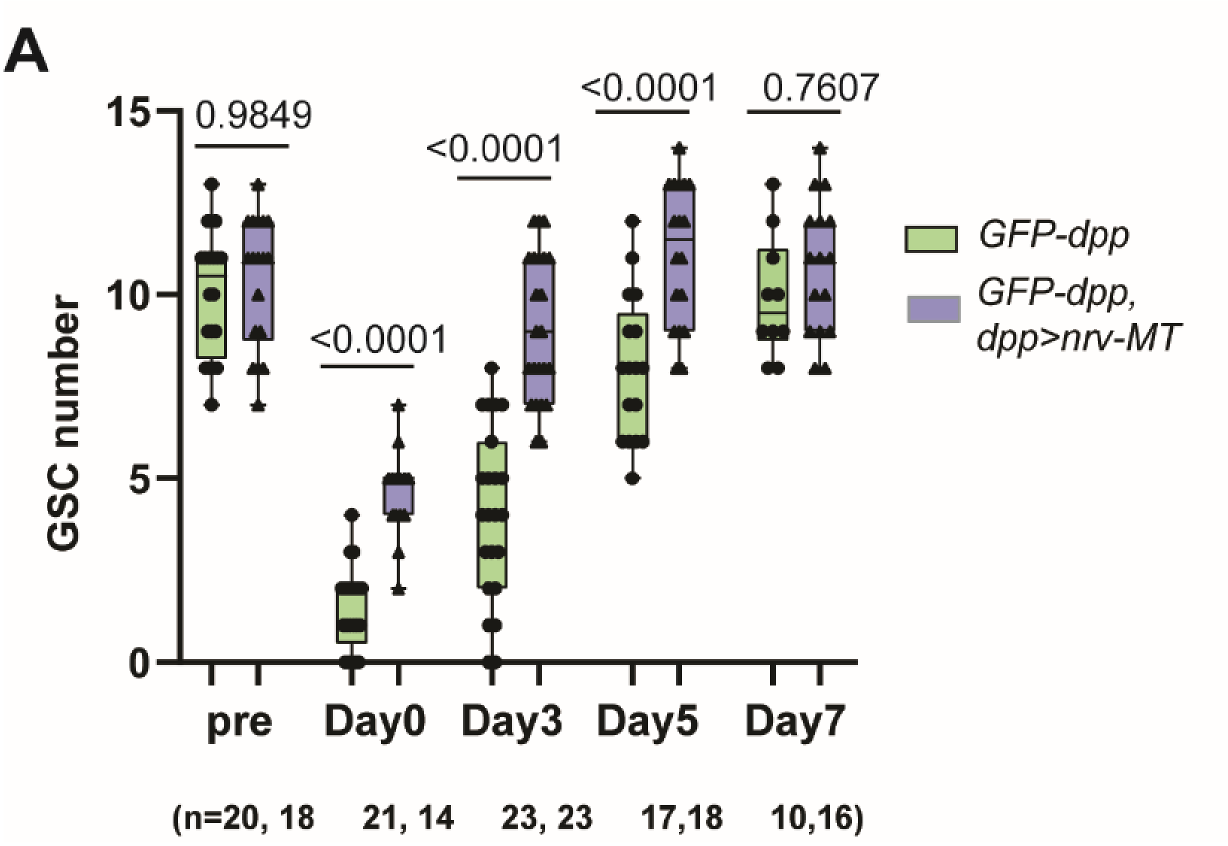
A
0.9849
<0.0001
<0.0001
<0.0001
0.7607
15
10
5
0
GSC number
GFP-dpp
GFP-dpp,
dpp>nrv-MT
pre
Day0
Day3
Day5
Day7
(n=20, 18 21, 14 23, 23 17,18 10,16)

Figure S3. Morphotrap using alternative genotypes shows similar effect on de-differentiation.

A) Changes in GSC number during recovery from forced differentiation of GSCs. For trapping Dpp, Nrv-MT was expressed under the control of the dppGal4 driver in homozygous *GFP-dpp* CRISPR knock-in background. Homozygous *GFP-dpp* CRISPR knock-in flies are viable and fertile and were used for the control. P-values were calculated by Šídák's multiple comparisons tests and provided on the graph. Fixed samples were used for scoring. “n” indicates the number of scored testes. Box plots show 25–75% (box), minimum to maximum (whiskers) with all data points. Numerical values are provided in Source Data file.

Figure S4

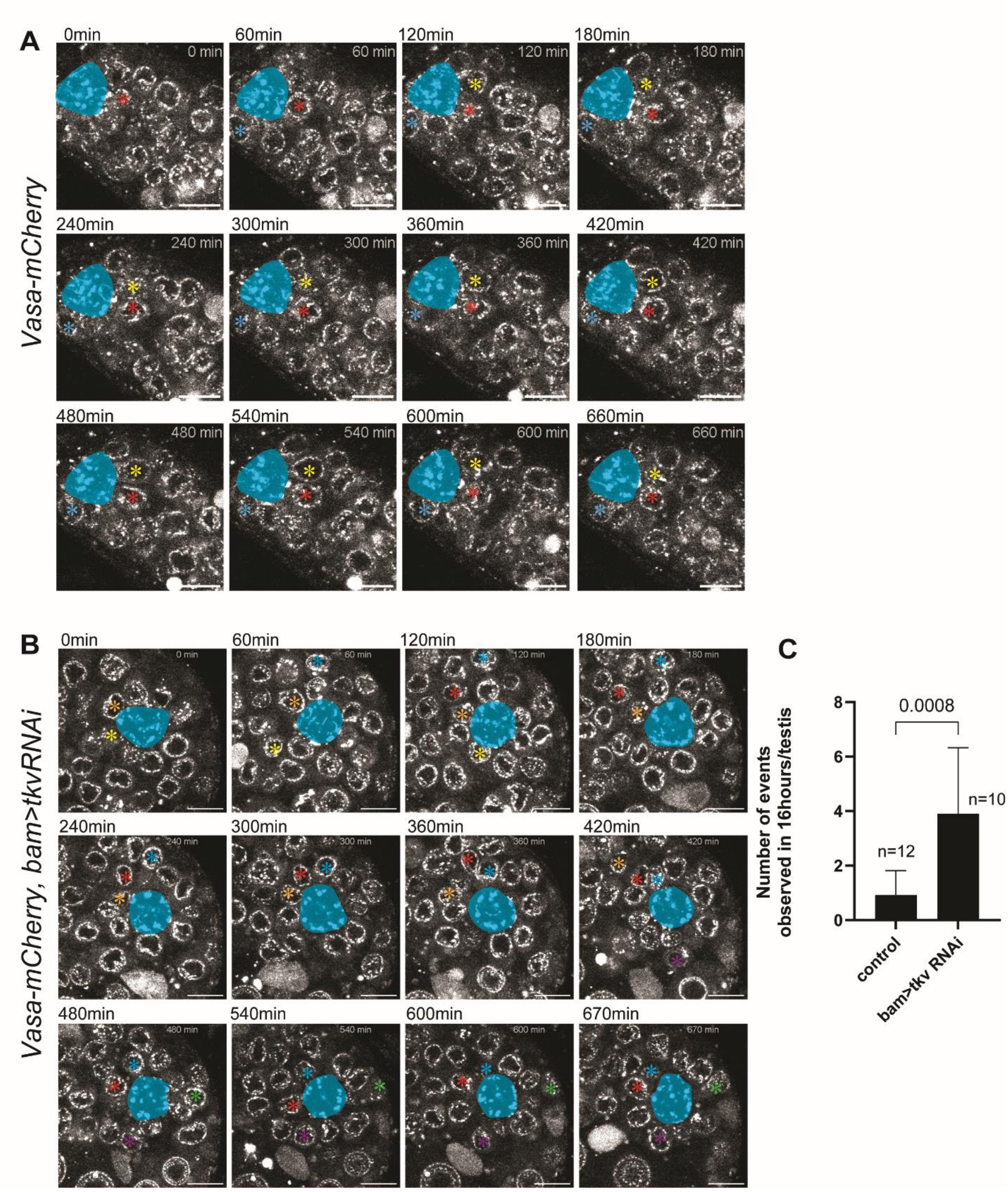
A
Vasa-mCherry
0min
0 min
60min
60 min
120min
120 min
180min
180 min
240min
240 min
300min
300 min
360min
360 min
420min
420 min
480min
480 min
540min
540 min
600min
600 min
660min
660 min
B
Vasa-mCherry, bam>tkvRNAi
0min
60min
120min
180min
240min
300min
360min
420min
480min
540min
600min
670min
C
Number of events observed in 16hours/testis
0.0008
n=12
n=10
control
bam>tkv RNAi
0
2
4
6
8

Figure S4. Live imaging of Tkv knock-down testis shows high frequency of non-asymmetric events.

A, B) Representative time-lapse live series of testis tip of indicated genotypes. Cells traced for entire imaging period are indicated by asterisks in each different color. In A, all marked GSCs stayed attached in the niche throughout the imaging period. In B, all marked cells show non-asymmetric behavior (yellow and orange cells leave from the hub, while other marked cells de-differentiate). Hub area is filled in turquoise blue. All scale bars represent 10 μm. Corresponding movies are provided as supplemental materials (Supplementary Movie S1 and Supplementary Movie S2). **C**) Number of non-asymmetric events observed in indicated genotypes in 16-hour imaging periods. Vasa-mCherry flies were used for the control. "n" indicates the number of time-lapse series analyzed. Data are means and standard deviation. The p-value was calculated by student-t-test. Numerical values are provided in Source Data file.

Figure S5

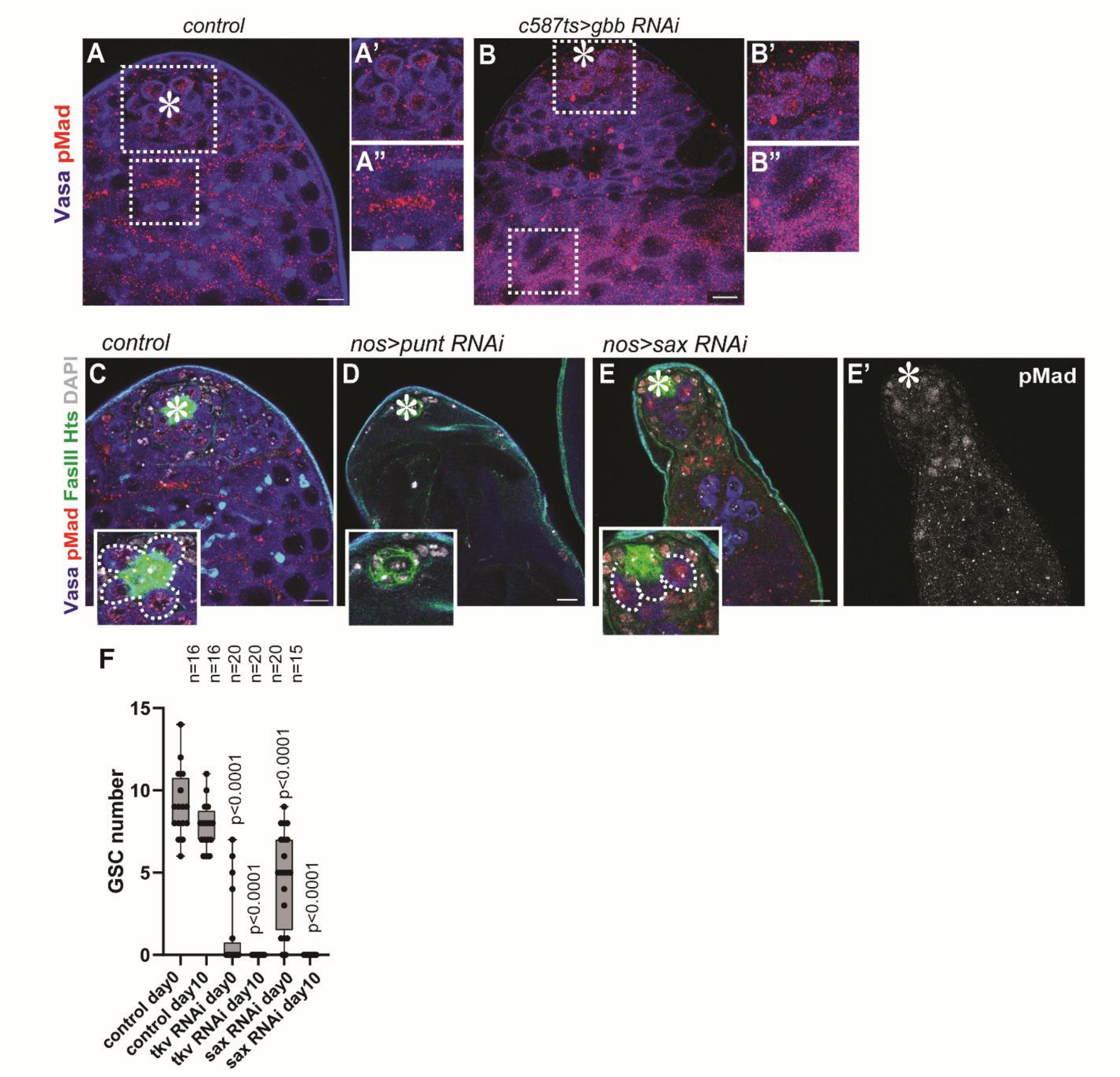
control
c587ts>gbb RNAi
A
A'
A''
B
B'
B''
Vasa pMad
control
nos>punt RNAi
nos>sax RNAi
C
D
E
E'
pMad
Vasa pMad FasIII Hts DAPI
F
n=16
n=16
n=20
n=20
n=20
n=15
GSC number
15
10
5
0
p<0.0001
p<0.0001
p<0.0001
p<0.0001
control day0
control day10
tkv RNAi day0
tkv RNAi day10
sax RNAi day0
sax RNAi day10

Figure S5. Sax and Punt show distinct effects on GSC maintenance.

A-B) Representative pMad staining images of testis tips with knock-down of *gbb* under the somatic cell specific driver, c587Gal4^{ts} after 4-days of temperature shift (29 degree). Squared regions in **A, B** are magnified in right panels. **A'**and **B'** show pMad signal in GSCs, which was intact in *gbb* RNAi testes. **A"**and **B"** show pMad signal in CCs, which was not detectable in *gbb* RNAi testes. **C-E)** Representative images of testis tips of no-Gal4 control (**C**) and with knock-down of *punt TRiP.GLV21066* (**D**) or sax *TRiP.HMJ02118* (**E**) under the control of nosGal4 driver. Insets show magnified region around the hub. Punt RNAi shows the testis without any Vasa positive germ cells. White broken lines encircle GSCs in inset **C** and **E,** showing GSCs with sax RNAi are pMad positive (**E**). **F**) GSC number in indicated age of testes of indicated genotypes. P-values were calculated by Šídák's multiple comparisons tests and provided on the graph. "n" indicates number of scored testes. Numerical values are provided in Source Data file. Scale bars represent 10 µm. Asterisks indicate approximate location of the hub. Fixed samples were used for all images.

Figure S6

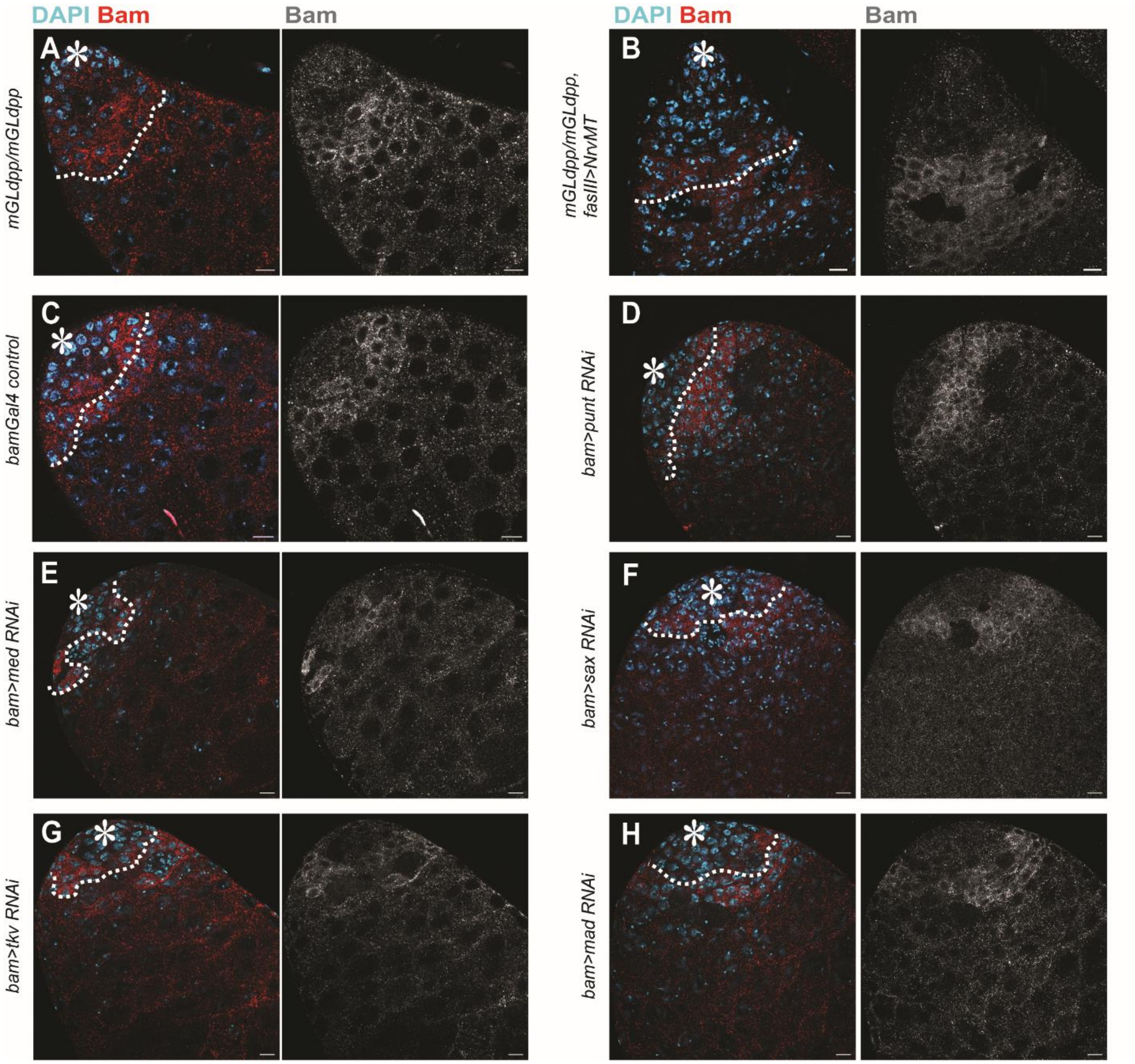
DAPI Bam
Bam
DAPI Bam
Bam
A
mGLdpp/mGLdpp
B
mGLdpp/mGLdpp, fasIII>NrvMT
C
bamGal4 control
D
bam>punt RNAi
E
bam>med RNAi
F
bam>sax RNAi
G
bam>tkv RNAi
H
bam>mad RNAi

Figure S6. BMP signal in SGs is required for timely upregulation of Bam.

A-H) Representative Bam staining images of indicated genotypes. Boundary between 8-cell SGs and 16-cell SGs are divided by white broken lines. Asterisks indicate approximate location of the hub. All scale bars represent 10 μm. Fixed samples were used for all images.

Legends for other supplemental materials

Supplementary Movie S1

A representative time-lapse movie of a testis tip (corresponding to Figure S4A). Time-interval: 10min. Scale bar: 10µm.

Supplementary Movie S2

A representative time-lapse movie of a testis tip (corresponding to Figure S4B). Time-interval: 10min. Scale bar: 10µm.

Source Data

Numerical values of all graphs are provided in this excel spreadsheet.

Supplementary Data1

Catalog number of commercial reagents used in this study is provided in this excel spreadsheet.
