# Supplementary material for: Diffusible fraction of niche BMP ligand safeguards stem-cell differentiation
Source: Nat Commun. 2024 Feb 7;15:1166. doi: 10.1038/s41467-024-45408-7 (PMC10850516; doi:10.1038/s41467-024-45408-7)
Supplement: Supplementary file 3 — Description of Additional Supplementary Files [file 41467_2024_45408_MOESM3_ESM.pdf]

Description of Additional Supplementary Files

File Name: Supplementary Data 1
Description: Catalog number of commercial reagents used in this study is provided in this excel spreadsheet.

File Name: Supplementary Movie 1
Description: A representative time-lapse movie of a testis tip (corresponding to Figure S4A). Time-interval: 10min. Scale bar: 10µm

File Name: Supplementary Movie 2
Description: A representative time-lapse movie of a testis tip (corresponding to Figure S4B). Time-interval: 10min. Scale bar: 10µm.
