# Supplementary material for: Diffusible fraction of niche BMP ligand safeguards stem-cell differentiation
Source: Nat Commun. 2024 Feb 7;15:1166. doi: 10.1038/s41467-024-45408-7 (PMC10850516; doi:10.1038/s41467-024-45408-7)
Supplement: Supplementary file 7 — Reporting Summary [file 41467_2024_45408_MOESM7_ESM.pdf]

nature portfolio

Corresponding author(s): Mayu Inaba, Shinya Matsuda

Last updated by author(s): Jan 7, 2024

Reporting Summary

Nature Portfolio wishes to improve the reproducibility of the work that we publish. This form provides structure for consistency and transparency in reporting. For further information on Nature Portfolio policies, see our Editorial Policies and the Editorial Policy Checklist.

Statistics

For all statistical analyses, confirm that the following items are present in the figure legend, table legend, main text, or Methods section.

n/a	Confirmed	
☐	☒	The exact sample size (*n*) for each experimental group/condition, given as a discrete number and unit of measurement
☐	☒	A statement on whether measurements were taken from distinct samples or whether the same sample was measured repeatedly
☐	☒	The statistical test(s) used AND whether they are one- or two-sided<br>*Only common tests should be described solely by name; describe more complex techniques in the Methods section.*
☒	☐	A description of all covariates tested
☐	☒	A description of any assumptions or corrections, such as tests of normality and adjustment for multiple comparisons
☐	☒	A full description of the statistical parameters including central tendency (e.g. means) or other basic estimates (e.g. regression coefficient) AND variation (e.g. standard deviation) or associated estimates of uncertainty (e.g. confidence intervals)
☐	☒	For null hypothesis testing, the test statistic (e.g. F, t, r) with confidence intervals, effect sizes, degrees of freedom and P value noted<br>*Give P values as exact values whenever suitable.*
☒	☐	For Bayesian analysis, information on the choice of priors and Markov chain Monte Carlo settings
☒	☐	For hierarchical and complex designs, identification of the appropriate level for tests and full reporting of outcomes
☒	☐	Estimates of effect sizes (e.g. Cohen's *d*, Pearson's *r*), indicating how they were calculated

Our web collection on statistics for biologists contains articles on many of the points above.

Software and code

Policy information about availability of computer code

Data collection	Confocal images were collected by a Zeiss LSM 800 Confocal microscope using ZEN software.
Data analysis	Confocal images were analyzed using ImageJ/Fiji software (version 2.1.0).<br>Statistical analysis and graphing were performed using GraphPad Prism software (version 9.2.0).<br>Illustrator (24.1.3) was used for figure preparation

For manuscripts utilizing custom algorithms or software that are central to the research but not yet described in published literature, software must be made available to editors and reviewers. We strongly encourage code deposition in a community repository (e.g. GitHub). See the Nature Portfolio guidelines for submitting code & software for further information.

Data

Policy information about availability of data

All manuscripts must include a data availability statement. This statement should provide the following information, where applicable:

- Accession codes, unique identifiers, or web links for publicly available datasets
- A description of any restrictions on data availability
- For clinical datasets or third party data, please ensure that the statement adheres to our policy

The data that support all experimental findings of this study are available within the paper and its Supplementary Information files. All imaging files will be available in the BioStudies database.

Research involving human participants, their data, or biological material

Policy information about studies with human participants or human data. See also policy information about sex, gender (identity/presentation), and sexual orientation and race, ethnicity and racism.

Reporting on sex and gender	N/A
Reporting on race, ethnicity, or other socially relevant groupings	N/A
Population characteristics	N/A
Recruitment	N/A
Ethics oversight	N/A

Note that full information on the approval of the study protocol must also be provided in the manuscript.

Field-specific reporting

Please select the one below that is the best fit for your research. If you are not sure, read the appropriate sections before making your selection.

☒ Life sciences ☐ Behavioural & social sciences ☐ Ecological, evolutionary & environmental sciences

For a reference copy of the document with all sections, see nature.com/documents/nr-reporting-summary-flat.pdf

Life sciences study design

All studies must disclose on these points even when the disclosure is negative.

Sample size	No statistical test was performed to predetermine sample size. We estimated following our previous works.
Data exclusions	No data were excluded.
Replication	Experiments were performed with at least 3 independent biological replicates. All results were reproduced at least 2 more times independently with similar results.
Randomization	The experiments were not randomized since all the experiments were categorized by distinct genotypes.
Blinding	Researchers were not blinded to data collection or analysis. Blinding was not possible since the experimental setup is easily identified.

Reporting for specific materials, systems and methods

We require information from authors about some types of materials, experimental systems and methods used in many studies. Here, indicate whether each material, system or method listed is relevant to your study. If you are not sure if a list item applies to your research, read the appropriate section before selecting a response.

Materials & experimental systems

n/a	Involved in the study	
☐	☒	Antibodies
☒	☐	Eukaryotic cell lines
☒	☐	Palaeontology and archaeology
☐	☐	Animals and other organisms
☒	☐	Clinical data
☒	☐	Dual use research of concern
☒	☐	Plants

Methods

n/a	Involved in the study	
☒	☐	ChIP-seq
☒	☐	Flow cytometry
☒	☐	MRI-based neuroimaging

Antibodies

Antibodies used	The primary antibodies used were as follows: rat anti-Vasa (RRID: AB_760351, 1:20; DSHB); mouse anti-Hts (1B1; RRID: AB_528070, 1:20; DSHB); mouse-anti-FasIII (RRID:AB_528238, 1:20, 7G10; DSHB); mouse anti-γ-Tubulin (GTU-88; RRID:AB_532292, 1:400; Sigma-Aldrich); Rabbit anti-pMad (RRID:AB_491015, 1:300; Cell Signaling Technology, Cat# 9516); Mouse anti-phospho-Histone H3 (Ser10),

	clone 3H10 (RRID:AB_477061; 1:200, Sigma-Aldrich); Rabbit anti-HA C29F4 (RRID:AB_1549585, 1:300, Cell Signaling Technology, Cat# 3724).
Validation	All antibodies were validated previously. Rat anti-Vasa (RRID: AB_760351, 1:20; DSHB); Mouse anti-Hts (1B1; RRID: AB_528070, 1:20; DSHB); Mouse-anti-FasIII (RRID:AB_528238, 1:20, 7G10; DSHB) have been validated in Drosophila gonads, and multiple papers are referenced on manufacturers website (https://dshb.biology.uiowa.edu/). Other antibodies used were validated in gonads or wing discs. Mouse anti-γ-Tubulin (PMID: 22357619), Rabbit anti-pMad (PMID: 26550827), Mouse anti-phospho-Histone H3 (Ser10), clone 3H10 (PMID: 29985130), Rabbit anti-HA C29F4 (PMID:20453847).

Animals and other research organisms

Policy information about studies involving animals; ARRIVE guidelines recommended for reporting animal research, and Sex and Gender in Research

Laboratory animals	Flies were raised on standard Bloomington medium (Lab express) at 25°C (unless temperature control was required). The following fly stocks were obtained from Bloomington stock center (BDSC); nosGal4 (BDSC64277); hs-bam (BDSC24636); tkv RNAi (BDSC40937); Nrv1 Morphotrap (lexAop-UAS-GrabFP.B.Ext.TagBFP, BDSC68173); mCD8-Morphotrap (lexAop-UAS-Morphotrap.ext.mCh, BDSC68170); medea RNAi:TRiP.GL01313 (BDSC43961); mad RNAi:TRiP.JF01264 (BDSC31316); sax RNAi:TRiP.HMJ02118 (BDSC42546); punt RNAi: TRiP.HMS01944 (BDSC39025); punt RNAi: TRiP.GLV21066 (BDSC35701); gbb RNAi:TRiP.HMS01243 (BDSC34898); tkv-CA (BDSC36537); UAS-GFP.dsRNA.R (BDSC44415); gbb-GFP.R (BDSC63055). yw (BDSC189) was used for wildtype. UAS-GFP-Mad (51), HA-dpp (22), UAS-HA-trap (22), HA-gbb (32) dppGal4 (22) lines are described elsewhere. FasIIIGal4 was obtained from DGRC, Kyoto Stock Center (A04-1-1 DGRC#103-948). GFP-dpp and mCherry-dpp (FBst0086273) CRISPR knock-in lines were kind gift from Thomas Kornberg and Ryo Hattori (21). pVas-Vasa-mCherry (FBtp0065762) (55), UAS-histone H3-GFP and bamGal4 on 3rd was kind gifts from Yukiko Yamashita.<br>Temperature shift was performed by culturing flies at room temperature and shifted to 29℃ upon eclosion for the 4 days before analysis. Combinations of Tub-Gal80ts (a gift from Cheng-Yu-Lee) with c587Gal4 (a gift from Yukiko M. Yamashita) were used.<br>For all crosses for obtaining mGL-dpp homozygous flies, transgenic allele containing dpp locus (pPA dpp 8391/X) (23) was introduced to assist embryonic expression and rescue semi-lethality. This transgene has been known to only rescue early development of dpp null mutant (23).
Wild animals	No wild animals were used in this study, only laboratory strains of Drosophila melanogaster.
Reporting on sex	This study focuses on Drosophila male germline stem cells, thus only male flies were used.
Field-collected samples	This study did not involve field-collected samples
Ethics oversight	No ethical approval or guidance is required for invertebrate model animals like Drosophila

Note that full information on the approval of the study protocol must also be provided in the manuscript.
